# Adapted yoga to improve physical function and health-related quality of life in physically-inactive older adults: a randomised controlled pilot trial

**DOI:** 10.1186/s12877-017-0520-6

**Published:** 2017-06-23

**Authors:** Garry A. Tew, Jenny Howsam, Matthew Hardy, Laura Bissell

**Affiliations:** 1 Department of Sport, Exercise and Rehabilitation, Northumbria University, Northumberland Building, Northumberland Road, Newcastle upon Tyne, NE1 8ST UK; 2 Yorkshire Yoga & Therapy Centre, Knaresborough, Harrogate, HG5 0TG UK

**Keywords:** Mind-body therapies, Randomised controlled trial, Aged, Physical fitness, Mental health

## Abstract

**Background:**

Yoga is a holistic therapy of expanding popularity, which has the potential to produce a range of physical, mental and social benefits. This trial evaluated the feasibility and effects of an adapted yoga programme on physical function and health-related quality of life in physically-inactive older adults.

**Methods:**

In this randomised controlled pilot trial, 52 older adults (90% female; mean age 74.8 years, SD 7.2) were randomised 1:1 to a yoga programme or wait-list control. The yoga group (*n* = 25) received a physical activity education booklet and were invited to attend ten yoga sessions during a 12-week period. The control group (*n* = 27) received the education booklet only. Measures of physical function (e.g., Short Physical Performance Battery; SPPB), health status (EQ-5D) and mental well-being (Warwick-Edinburgh Mental Well-being Scale; WEMWBS) were assessed at baseline and 3 months. Feasibility was assessed using course attendance and adverse event data, and participant interviews.

**Results:**

Forty-seven participants completed follow-up assessments. Median class attendance was 8 (range 3 to 10). At the 3-month follow-up, the yoga group had a higher SPPB total score compared with the control group (mean difference 0.9, 95% confidence interval [CI] -0.3 to 2.0), a faster time to rise from a chair five times (mean difference − 1.73 s, 95% CI −4.08 to 0.62), and better performance on the chair sit-and-reach lower-limb flexibility test (mean difference 5 cm, 95% CI 0 to 10). The yoga group also had superior health status and mental well-being (vs. control) at 3 months, with mean differences in EQ-5D and WEMWBS scores of 0.12 (95% CI, 0.03 to 0.21) and 6 (95% CI, 1 to 11), respectively. The interviews indicated that participants valued attending the yoga programme, and that they experienced a range of benefits.

**Conclusions:**

The adapted yoga programme appeared to be feasible and potentially beneficial in terms of improving mental and social well-being and aspects of physical function in physically-inactive older adults. An appropriately-powered trial is required to confirm the findings of the present study and to determine longer-term effects.

**Trial registration:**

ClinicalTrials.gov NCT02663726.

## Background

Older adults (i.e., aged ≥60 years) who are physically inactive are at increased risk of falls, functional limitations, disability, and mental health problems [1–4]. Encouragingly, there is evidence that various physical activity interventions can elicit meaningful improvements in physical function and health-related quality of life in older people. Effective programmes have included aerobic exercise [5], progressive resistance training [6] and Tai Chi [7]. Yoga is an alternative approach to improving fitness and health outcomes in older adults.

In the West, the word ‘Yoga’ is the general term used for the practice of ‘Hatha Yoga’. Hatha Yoga is a centuries-old health and well-being system from India that involves a combination of physical postures or poses (*asana*), breathing exercises (*pranayama*), integrated breath-movement sequences, relaxation, and concentration/meditation. Many styles of Hatha Yoga have developed over the past few decades due to the global expansion of the teaching conducted by influential instructors such as Iyengar, Desikachar (Viniyoga), Pattabhi Jois (Astanga), Sivananda and Bikram (Hot Yoga). The differences are subtle, but the traditional physical poses (*asana*) of Hatha Yoga are the same. All Hatha Yoga classes require participants to hold and move between various stationary positions with the goal of developing strength, balance and flexibility. To ensure a total body workout, a mixture of standing, seated, kneeling, supine, and prone stationary positions are used, with transitions incorporating forward bends, back bends, side bends, twists, inversions and balances. All Hatha Yoga styles honour the importance of breathing exercises, mental concentration/meditation and relaxation.

The proposed benefits of regular Yoga practice are many and varied, including increases in muscular strength, flexibility and balance, reduced stress, anxiety and depression, and an enhancement of overall well-being and quality of life [8]. Interestingly, the findings of a systematic review of 16 studies (*n* = 649) [9] and a more recent trial of 118 participants [10] indicate that Yoga (of various types) may provide greater improvements in physical functioning and self-reported health status than conventional physical activity interventions in elderly people. However, the previous studies had limitations, including small sample sizes, a single Yoga teacher delivering the programme, and short-term follow-up. Furthermore, none of the included studies had been conducted in the United Kingdom.

In 2009, the British Wheel of Yoga (BWY) Gentle Years Yoga© programme was developed in North Yorkshire, England to cater specifically for the needs of older people with age-related conditions such as osteoarthritis, hypertension, dementia, and sensory impairment. Adaptations to challenging Hatha Yoga poses were developed so that inactive older adults with comorbidities and physical limitations could safely participate whilst still reaping the health and well-being benefits of Hatha Yoga. This pragmatic randomised controlled pilot trial represents the first formal evaluation of this programme. Therefore, the aim of this study was to investigate the feasibility of the British Wheel of Yoga (BWY) Gentle Years Yoga© programme in physically-inactive older adults with various comorbidities, as well as its effects on physical function and health-related quality of life.

## Methods

### Trial design

In this randomised controlled trial, participants were assigned to one of two groups: Yoga programme or wait-list control. Recruitment occurred between February and March 2016, and data collection was performed at the Yorkshire Yoga & Therapy Centre between March and July 2016. The Northumbria University Faculty of Health and Life Sciences Research Ethics Committee approved the study (reference HLSGT180116). The trial was registered with the ClinicalTrials.gov (reference NCT02663726).

### Setting and participants

The trial was conducted at a Yoga centre, two community-based facilities, and one care home in North Yorkshire, United Kingdom. Participant recruitment was undertaken via advertising in local newspapers, websites, and newsletters from local community organisations. Individuals were eligible if they were aged 60 years or older, and were willing and able to attend the assessment sessions and Yoga classes. Exclusion criteria included having a medical condition that precludes exercise [11] (e.g., unstable cardiac disease, uncontrolled hypertension, and uncontrolled metabolic diseases), having major surgery scheduled within 3 months of the baseline visit, current participation in >90 min per week of purposeful exercise (self-reported average over the past year), and participation in another clinical trial for which concurrent participation was deemed inappropriate. The presence or absence of these factors was determined by the research team during the first telephone contact with prospective participants. Written, informed consent was obtained for all participants prior to the baseline assessments.

As this was a pilot study, no formal sample size calculation was performed [12]. Instead, we aimed to recruit at least 40 participants within the 2-month recruitment period. We believed this to be a feasible target, and one that would provide useful information for the design of a future definitive randomised controlled trial.

### Baseline questionnaire

At the baseline visit, all participants completed a questionnaire that included questions relating to their age, sex, ethnicity, lifestyle habits, employment status, and current medications. In addition, participants were asked to indicate which of 27 comorbidities they suffered from; the list being based on work by Bayliss et al. [13].

### Randomisation and interventions

Following completion of baseline assessments, which were all conducted on the same day, participants were randomly allocated in a ratio of 1:1 to Yoga intervention or wait-list control. The randomisation sequence was computer-generated by an investigator who was not involved in the recruitment process and was stratified by site, with one block per site.

All participants received an education booklet about physical activity for older adults [14]. The intervention group was also offered a Yoga programme, free of charge. The wait-list control group were offered the same Yoga programme, free of charge, after the 3-month follow-up assessment.

### BWY Gentle Years Yoga© programme

Eight experienced Yoga teachers were recruited and trained for the study. All eight attended free practical training over three consecutive days in BWY Gentle Years Yoga© methods which included Safeguarding Vulnerable Adults training and a Dementia Friends information session. One trainee teacher lived in London and another in York and therefore they did not become teachers in the study which took place in Harrogate District of North Yorkshire, England. All teachers in the study taught the same form of Yoga as outlined in the BWY Gentle Years Yoga© training material. One teacher was selected to deliver each of the four courses, leaving three teachers spare to serve as back-up. Treatment fidelity was assessed through observation of each teacher’s class teaching on two separate occasions by JH, with assessments quality assured by LB. In addition to the three practical days of training (24 guided learning hours), the six teachers did a minimum of 46 h of home study, including assessed written work and exams.

The four Yoga courses were all delivered at different sites: one Yoga centre, two community centres and one care home for residents diagnosed with dementia and other co-morbidities. One of the community centres was located in one of the 20% most-deprived neighbourhoods in England.

**Fig. 1:**
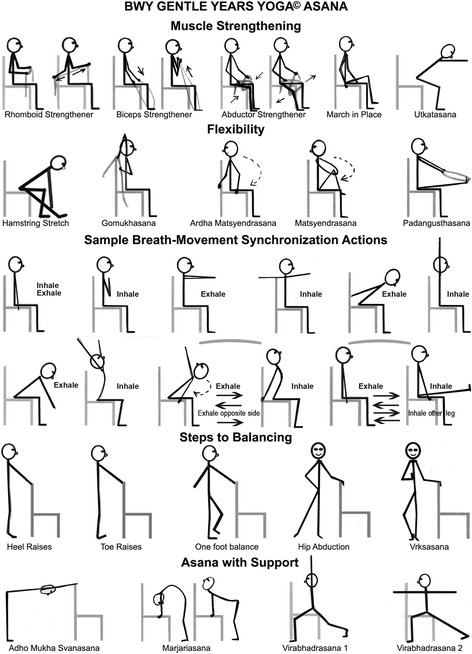
Sample of chair-based poses that were used in the adapted yoga programme

Each course involved ten 75-min, group-based classes delivered across a 12-week period (approximately one class per week). The programme introduced participants to the foundational elements of Yoga adapted appropriately for older adults, including *asana*, *pranayama*, relaxation techniques, mental focus, and philosophy. Classes consisted of an introduction to the weekly theme, pain-relieving or settling-in relaxing poses, a programme of seated and standing practices, educative postural advice, breath work, concentration activities, and 5 to 15 min of relaxation. Examples of the seated poses used are shown in Fig. 1. Poses targeted stiff, weak, and untrained areas of the whole body, with the intention of improving mobility, strength, and posture and reducing pain. Later classes featured postures that built on previous weeks (in accordance with the training principle of progressive overload), with a key aim of increasing participants’ ability and confidence to perform activities of daily living (e.g. climbing stairs, getting out of a chair).

During the supervised classes, the teachers modified the practices so that each individual could adopt a safe variation of the poses and sequences that would not compromise their health. For example: when the class was performing a forward bend, individuals with osteoporosis were instructed to flex only at the hip and to avoid flexion of the spine; people with hypertension or cardiac conditions learned to modify the angle of the forward bend so that the head was never lower than the heart; people with replacement knees or hips were taught how to perform *asana* such as *utkatasana* (or sit-to-stand modification) and *virabhadrasana II* (seated warrior 2) without jeopardising the artificial joint, and; people with dementia or suspected cognitive impairment were given tools to help them to remember sequences, such as performing actions while singing well-known songs from childhood. The following bullet points summarise how the adapted Yoga classes differ from standard Hatha Yoga classes:

- For the most part, participants are seated on chairs, and when standing, they use the chair or other aids for support;
- The classes do not use supine, semi-supine or prone *asana*; instead, the key elements of traditional supine/prone *asana* are integrated into seated or standing postures;
- The classes hold static *asana* (isometric postures) for a shorter length of time, especially those that could cause more pronounced acute increases in blood pressure;
- The physical set up of the class has been adapted to suit people with sensory impairment; specifically, participants being relatively close to the instructor, lighting levels being higher, the colour of equipment being in contrast to that of the walls and floor, and no music played during instruction;
- The pace and overall structure of the class allows greater time for recovery from the more intense activities (e.g. by having a simple breathing practice follow a more-challenging *asana*);
- Instructions are in short, single-point sections with time allowed for information processing to facilitate understanding in individuals with cognitive impairment;
- Longer warm-up and overall slower pace, making it safer for older adults and at a level where they can work without feeling ‘left behind’ or ‘too old for Yoga’, or having their self-confidence eroded;
- Breathing practices avoid retention, as this is contraindicated for individuals with hypertension;
- Mobilisation, *asana*, and concentration exercises (*dharana*) are incorporated that specifically focus on balance and co-ordination.

Once the teachers were satisfied that the participants knew how to adapt the exercises for their medical conditions, self-practice sheets were distributed and the participants were encouraged to practice selected Yoga activities at home for 10–20 min on most days. As the supervised work in class became progressively more challenging, students were given new information sheets that allowed them to develop their home Yoga routine. There were three information sheets in total, and these were typically distributed in weeks 1, 3 and 6.

### Study measures

Outcomes were measured before randomisation and 3 months after randomisation. Assessors were blinded to group assignment. The primary outcome measures were the total score on the Short Physical Performance Battery (SPPB), and performance on the individual components of the SPPB: standing balance, chair sit-to-stand, and 4-m walking time. The SPPB is a functional performance measure that depends on leg strength and balance [15, 16], which were targets of the Yoga programme.

#### Short Physical Performance Battery (SPPB)

The SPPB combines data from standing balance, time to rise and stand from a seated position 5 times, and time to walk 4 m at a usual pace. Individuals receive a score of 0 for each task they are unable to complete. Scores of 1 to 4 are assigned for remaining tasks, according to established methods [15, 16]. Scores are then summed to obtain a total score ranging from 0 to 12 [15, 16].

For the standing balance component, participants are asked to hold 3 increasingly difficult standing positions for 10 s each: the side-by-side stand, semi-tandem stand (standing with feet parallel and the heel of one foot touching the base of the first toe of the opposite foot), and the full-tandem stand (standing with one foot directly in front of the other) [15, 16]. Scores range from 0 (unable to hold the side-by-side stand for 10 s) to 4 (able to hold the full-tandem stand for 10 s) [15, 16].

For the chair sit-to-stand component, participants sit in a straight-backed chair with arms folded across their chest and stand 5 times consecutively as quickly as possible. Time to complete 5 chair rises is measured [15, 16]. Scores range from 0 (unable to complete 5 chair rises within 60 s) to 4 (able to complete 5 chair rises in ≤11.1 s) [15, 16].

For the walking component, participants are asked to complete a timed 4-m walk at a usual pace. The lowest time (quickest walk) from two valid attempts was recorded at baseline and follow-up. Scores range from 0 (unable to complete) to 4 (able to complete in <4.82 s) [17].

The following variables from the SPPB were analysed: total SPPB score; standing balance time, calculated as the sum of time able to stand in the three positions, up to a maximum of 30 s; chair sit-to-stand time, measured as the time (in seconds) to complete 5 chair rises, and; the time (in seconds) to walk 4 m at a usual pace.

#### Secondary outcome measures

Secondary outcome measures included body mass and stature (for the calculation of body mass index), waist circumference, resting systolic and diastolic blood pressure (A&D TM-2655P, PMS Instruments Ltd., Berkshire, UK), the EuroQol EQ-5D-5L health index [18], the Warwick-Edinburgh Mental Well-being Scale (WEMWBS) [19], upper- and lower-body flexibility using the back-scratch and chair sit-and-reach components of the Senior Fitness Test [20], respectively, and adverse events.

The EQ-5D-5L is a simple, self-administered measure of health status that comprises two parts. The first part comprises five health dimensions – mobility, self-care, usual activities, pain/discomfort and anxiety/depression – with five levels of severity for each dimension [18]. Together, the five responses represent a ‘health state’, which can be converted using a standard algorithm to produce a single health state utility score [21]. The second part asks the respondent to assess their health ‘today’ on a visual analogue scale (VAS) of 0 to 100.

The WEMWBS is a 14-item questionnaire that uses a 5-point Likert scale to give a score of one to five for item and a total score ranging 14 to 70 – a higher WEMWBS score indicates a higher level of mental well-being [19].

#### Intervention acceptability

The acceptability of the study design and Yoga programme was assessed using class attendance rates and participant feedback via telephone interviews conducted within a 2-week period following the 3-month assessment. The participant interviews lasted up to 20 min and covered perceived benefits and negative consequences from participating in the study, feedback regarding specific design features of the study (including the Yoga programme and assessment procedures), and perceptions of barriers and facilitators to intervention participation. Audio recordings of the interviews were subsequently transcribed, before two of the authors (GT and LB) used a thematic analysis approach to summarise the data. This involved listening to the audio files and reading the transcripts several times to become familiar with the data, pooling quotes from different participants that related to the same part of the interview topic guide, and producing a summary of the findings for each part of the interview topic guide.

### Statistical analysis

The effect of the intervention was evaluated using an analysis of covariance model. The 3-month outcome was the dependent variable and trial arm (intervention and control) was the independent variable. The baseline value of the outcome was included as a covariate [22], with study site as a random effect [23]. The analyses were done on an intention-to-treat basis, including only those participants with both baseline and follow-up data available (i.e., complete case analysis). The treatment effect (intervention minus control) is presented with its 95% confidence interval (CI). Analyses were conducted using IBM SPSS Statistics Version 22 (IBM United Kingdom Limited, Hampshire, UK).

## Results

**Fig. 2:**
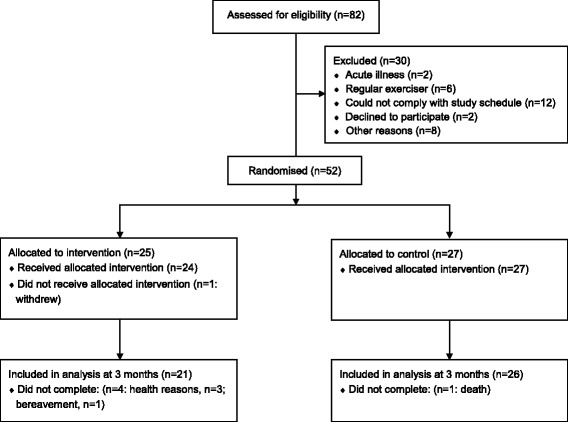
The flow of participants through the trial

A total of 82 people were screened, and 52 (63.4%) were randomised among the four centres: 25 to Yoga and 27 to wait-list control (Fig. 2). Forty-seven (90%) of the participants were female and the mean age was 74.8 years (SD 7.2). The participants were all white, and the majority were retired (92%) and living in a community dwelling (88%). Participants often had multiple comorbidities (range 0 to 6), which included osteoarthritis (*n* = 20), hypertension (*n* = 12), depression/anxiety (*n* = 11), hypercholesterolemia (*n* = 7), rheumatoid arthritis (*n* = 6), asthma (*n* = 6), dementia (*n* = 4), osteoporosis (*n* = 4), cancer (*n* = 4), and cerebrovascular disease (*n* = 4). The participants in the two groups had similar baseline characteristics (Table 1), although there was a higher proportion of previous smokers in the control group (60% versus 33%).

**Table 1:** Demographic and baseline characteristics of participants

Characteristic	Intervention (*n* = 25)	Control* (*n* = 27)
Age, years	73.8 (6.5)	75.7 (7.9)
Female sex, n (%)	25 (100)	22 (81)
White ethnicity, n (%)	25 (100)	27 (100)
Employment status, n (%)		
Employed full-time Employed part-time Retired	0 (0) 3 (12) 22 (88)	1 (4) 0 (0) 26 (96)
Living situation, n (%)		
Community dwelling Care home resident	22 (88) 3 (12)	24 (89) 3 (11)
Smoking status, n (%)		
Current Previous Never	0 (0) 9 (36) 16 (64)	0 (0) 15 (58) 11 (42)
Total medications, median (range)	3 (0–8)	4 (0–12)
Comorbidities, n (%)		
0 1 2–3 4 or more	4 (16) 10 (40) 9 (36) 2 (8)	5 (19) 5 (19) 9 (33) 8 (29)
Physical characteristics		
Body mass, kg Stature, cm Body mass index, kg/m^2^ Waist circumference, cm Heart rate, beats/min Systolic blood pressure, mmHg Diastolic blood pressure, mmHg	73.2 (14.1) 159.5 (5.8) 28.8 (5.7) 94.2 (13.0) 78 (16) 150 (30) 84 (17)	79.3 (19.9) 159.4 (8.9) 31.0 (6.3) 99.3 (12.4) 80 (11) 141 (22) 79 (11)
Physical function		
SPPB total score^a^ Standing balance^b^, s Sit to stand^b^, s Four-metre walk time^b^, s Chair sit-and reach^a^, cm Back scratch^a^, cm	9.7 (1.9) 27.65 (4.32) 16.51 (7.11) 4.07 (0.78) −5 (9) −10 (12)	8.2 (2.3) 26.96 (6.03) 22.59 (11.90) 5.81 (4.46) −8 (14) −18 (17)
Questionnaires		
EQ-5D utility index^a^ EQ-VAS^a^ WEMWBS^a^	0.74 (0.16) 71 (13) 50 (11)	0.64 (0.20) 64 (17) 52 (8)

Data are presented as mean (SD) unless otherwise stated

*Data were missing for smoking status (*n* = 1), all physical characteristics (*n* = 1), all physical function tests (*n* = 1) and all questionnaire scores (*n* = 1)

^a^Higher score is better; ^b^Lower score is better

*EQ-VAS* EuroQol Visual Analogue Scale, *SPPB* Short Physical Performance Battery, *WEMWBS* Warwick-Edinburgh Mental Well-being Scale

The 52 participants heard about the study in the following ways: 22 (42.3%) via newspaper articles/advertisements; 11 (21.2%) via taster sessions at an Arthritis Care meeting, community centres, and a residential care home; 10 (19.2%) via a Yorkshire Yoga E-newsletter and friends/relatives of people at Yorkshire Yoga; 8 (15.4%) via leaflets delivered door-to-door in areas with a high concentration of older people, and; 1 (1.9%) via information displayed in a GP surgery.

### Participant retention and intervention attendance

Four Yoga participants did not complete the follow-up assessments: three withdrew because of health problems that were unrelated to the study (eye surgery [*n* = 1], liver and kidney problems [*n* = 1], and “unwell”/bed-bound [*n* = 1]) and one withdrew due to a family bereavement. These participants had completed 0, 3, 5, and 6 Yoga sessions, respectively, before withdrawing. One control participant died before the follow-up assessment. The death was unrelated to the study.

Of the 21 Yoga participants who completed the study, median class attendance was 8 (range 3 to 10). Eighteen (86%) attended at least 5 of the 10 classes, and 14 (67%) attended at least 8 of the 10 classes. All of the treatment fidelity checks indicated that the Yoga sessions were delivered to the standards set in the initial teacher training.

### Effect of intervention on outcome measures

The Yoga group had better self-reported health status and mental well-being at 3 months than the control group (Table 2). The adjusted mean EQ-5D utility, EQ-Visual Analogue Scale, and WEMWBS scores were 0.12 (95% CI, 0.03 to 0.21), 17 (8 to 26), and 6 (1 to 11) points higher in the Yoga group at 3 months, respectively. Lower body flexibility (chair sit-and-reach test) was also better in the Yoga group at 3 months (mean difference 5 cm, 0 to 10 cm). All aspects of physical function also appeared to be superior in the Yoga group at 3 months. Table 2 reveals that most of the 95% confidence intervals lie on the positive side, suggestive of beneficial effects. However, the confidence intervals also reveal that the true population effects could range from trivial negative (harmful) effects of 0.07 to 0.18 SD to moderate beneficial effects of 0.44 to 0.78 SD. There were unclear effects on body mass index, waist circumference and blood pressure; however, resting heart rate was lower in the Yoga group at 3 months (mean difference 6 beats/min, 1 to 11). There was one non-serious adverse event, which was probably related to the Yoga programme. Here, the participant reported that specific exercises aggravated her existing lower back pain during the first couple of sessions, but that this problem soon subsided and did not occur again for the remainder of the course.

**Table 2:** Outcome data for completers at baseline and 3 months

Characteristic	Intervention (*n* = 21)		Control (*n* = 26)		Adjusted mean difference between groups at 3 months* (95% CI)
	Baseline	3 months	Baseline	3 months	
Physical characteristics					
Body mass, kg Body mass index, kg/m^2^ Waist circumference, cm Heart rate, beats/min Systolic blood pressure, mmHg Diastolic blood pressure, mmHg	74.4 (14.8) 29.4 (6.0) 96.2 (12.6) 79 (17) 153 (32) 87 (17)	74.2 (15.2) 29.3 (6.1) 94.5 (12.7) 74 (14) 142 (22) 82 (13)	79.3 (19.9) 31.0 (6.3) 99.3 (12.4) 80 (11) 141 (22) 79 (11)	78.0 (19.0) 30.5 (6.0) 98.7 (12.4) 80 (11) 138 (24) 75 (13)	1.0 (−0.4 to 2.5) 0.4 (−0.2 to 0.9) −0.8 (−4.0 to 2.3) −6 (−11 to −1) −3 (−14 to 8) 3 (−5 to 11)
Physical function					
SPPB total score^a^ Standing balance^a^, s Sit to stand^b^, s Four-metre walk time^b^, s Chair sit-and reach^a^, cm Back scratch^a^, cm	9.5 (1.9) 27.51 (4.55) 16.45 (6.63) 4.10 (0.83) −7 (9) −11 (13)	10.0 (1.8) 29.00 (3.08) 14.64 (4.52) 4.04 (0.87) −2 (10) −7 (12)	8.2 (2.3) 26.96 (6.03) 22.59 (11.90) 5.81 (4.46) −8 (14) −18 (17)	8.2 (2.2) 27.44 (4.82) 19.63 (8.10) 5.28 (1.84) −8 (13) −16 (17)	0.9 (−0.3 to 2.0) 1.50 (−0.98 to 3.97) −1.73 (−4.08 to 0.62) −0.63 (−1.47 to 0.21) 5 (0 to 10) 2 (−2 to 7)
Questionnaires					
EQ-5D utility index^a^ EQ-VAS^a^ WEMWBS^a^	0.72 (0.16) 71 (13) 49 (12)	0.81 (0.12) 83 (11) 56 (9)	0.64 (0.20) 64 (17) 52 (8)	0.63 (0.22) 63 (17) 52 (10)	0.12 (0.03 to 0.21) 17 (8 to 26) 6 (1 to 11)

Data are presented as unadjusted mean (SD) unless stated otherwise

*Intervention minus control; Adjustment for site and baseline score

^a^Higher score is better; ^b^Lower score is better

N.B. on ANCOVA assumptions: (i) The normality assumption appeared to be violated for standing balance. Data on the proportion of participants from each group who achieved a highest possible score on this test are described in the discussion; (ii) Participant 1303 from the control group appeared to be a statistical outlier for the sit-to-stand, four-metre walk, back scratch and EQ-5D utility index variables. Sensitivity analyses excluding this individual from the data set showed trivial effect on the effect estimates (data not presented); (iii) Levene’s test of homogeneity of variance was significant (*P* < 0.05) for the SPPB, sit-to-stand and back scratch variables. Mann-Whitney U tests on follow-up scores produced *P* values of 0.009, 0.009 and 0.059 for these variables, respectively; (iv) homogeneity of regression slopes was verified for all variables except WEMWBS. A Mann-Whitney U test on follow-up scores produced a *P* value of 0.069 for this variable

ANCOVA, analysis of covariance; CI, confidence interval; EQ-VAS, EuroQol Visual Analogue Scale; SPPB, Short Physical Performance Battery; WEMWBS, Warwick-Edinburgh Mental Well-being Scale

### Interview feedback

Twenty (95%) of the 21 Yoga participants who completed the study also completed an exit interview. The one person who did not complete an interview was uncontactable despite numerous attempts. Eighteen people (90%) reported enjoying the Yoga programme and all 20 people stated the study procedures were acceptable and that they would recommend a study like this to other people they know. One person thought that the Short Physical Performance Battery was too easy, such that it was hard to see improvement on those tests, and one other person suggested that it may have been useful to include some type of walking endurance test. Fourteen people (70%) had paid to attend the next BWY Gentle Years Yoga© course, and three other people (15%) said that they were going to continue doing the home-based Yoga exercises. Reasons for people (*n* = 3) not continuing with Yoga included preference for other forms of exercise (*n* = 2) and that the classes were too easy (*n* = 1).

The interviewees reported a range of physical, mental and social benefits from participating in the Yoga programme (Table 3). The most commonly cited physical benefits included improved physical function (e.g., improved chair rising and walking ability) (*n* = 10), improved flexibility (*n* = 10) and reduced pain (*n* = 4). Cited mental health benefits included stress-relieving/calming effects (*n* = 7), improved mood (*n* = 2) and a reduced frequency of panic attacks (*n* = 1). Thirteen people (72%) indicated that they liked the social interaction that the group exercise classes provided. Furthermore, many of the participants explicitly stated that having other peoples’ company was one of the main benefits of attending the class, and that they had developed new friendships with other class attendees.

**Table 3:** Selected quotes indicating participants’ perceived physical, mental and social benefits of yoga participation

Physical benefits
*“I am more mobile and my back doesn’t hurt as much as it used to”* (1004, 74 years) *“I do feel more flexible … my legs are a little bit stronger … I’ve got bad shoulders, but they do actually feel a lot better.”* (1011, 68 years) *“my shoulder has greatly improved with the yoga … it’s not painful and the mobility is much better”* (1103, 67 years) *“taught me how to breathe with my asthma … sometimes I have trouble breathing and she [the instructor] taught me how to breathe through it”* (1103, 67 years) *“I got more movement in my shoulder … my hip movement became better … it’s easier going up and down stairs”* (1109, 68 years) *“I feel that I’ve got more range of movement, especially in my neck and mostly in my legs and shoulders … I can also bend forwards more easily … I’m sitting better as well.”* (1111, 69 years) *“I just got rid of the aches and I feel that I can use my fingers better and also my toes … I thought my walking was faster and my balance was easier”* (1404, 77 years) *“I have found more energy”* (1415, 83 years)
Mental benefits
*“you know the breathing … I do that sometimes if I’m feeling a bit stressed or down … I think it does help”* (1011, 68 years) *“I’m a bit prone to panic attacks, and I’ve not any for a few months now, so maybe the yoga’s helped … so that’s a plus point”* (1012, 67 years) *“I was a lot calmer from coming back after the yoga”* (1103, 67 years) *“I can lay in bed and take different breathing exercises, which relaxes me, calms me down.”* (1109, 68 years) *“My mood is better, I think it benefits your mood”* (1411, 65 years) *“I sit sometimes and do some of the actions, and I think it helps me when I start feeling depressed and missing [deceased husband’s name], it helps me to relax … I think physical movement helps your mental processes”* (1412, 79 years) *“it calmed my mind, and I could think and not feel sad … it relieved the stress and sadness”* (1412, 79 years) *“I like all the exercises and the breathing helps me feel calmer”* (1302, care home resident, 85 years)
Social benefits
*“you make so many friends … you have another circle of friends so that is beneficial”* (1004, 74 years) *“we had a laugh, a cup of tea and a chat afterwards”* (1011, 68 years) *“we allowed ourselves to have a little chat and a laugh, whilst still getting the work done”* (1012, 67 years) *“we all stayed for a chatter afterwards, and that’s very nice”* (1103, 67 years) *“I met somebody who I knew from before but didn’t know very well … we conjured up a little relationship. A lot of people don’t see a lot of people, so for some people it [the social aspect] is an added bonus.”* (1105, 71 years) *“It was nice getting to meet other people, and I looked forwards to going and meeting them.”* (1111, 69 years) *“meeting different women – was very important at my stage of life”* (1412, 79 years) *“it’s nice doing things with other people when you live alone … I enjoyed the company”* (1415, 83 years)

## Discussion

This trial found that the BWY Gentle Years Yoga© programme, when delivered once a week for 10 weeks, is safe, feasible, and acceptable for physically-inactive older adults with a broad range of comorbidities. The programme also led to improvements in health status, mental well-being and physical function at 3 months.

In this study, the 5-level EQ-5D and WEMWBS were used to assess health status and mental well-being, respectively. Although there is no consensus, changes in the EQ-5D utility index and WEMWBS of 0.10 [24] and between 3 and 8 points [25], respectively, have been recommended as clinically important. In this trial, we observed that scores on these questionnaires were, on average, 0.12 and 6 points higher, respectively, in the Yoga group at 3 months. These potentially-important changes are consistent with the findings of a meta-analysis of 422 older adults published in 2012 [9], which showed moderate beneficial effects of Yoga (versus other exercise interventions) on the physical and mental component scores of the SF-36 health survey (standard mean differences of 0.65 [0.02 to 1.28] and 0.66 [0.10 to 1.22], respectively). The small-to-moderate improvements we observed in physical function outcomes (e.g., lower-body flexibility, standing balance, sit-to-stand), are also in accordance with the results of a recent systematic review [26], which reported standardised mean differences for the effect of Yoga (versus control) on balance of 0.40 (0.15 to 0.65; 6 trials, *n* = 307), and on mobility of 0.50 (0.06 to 0.95; 3 trials, *n* = 225). That we did not see larger between-group differences for some aspects of physical function might be due to some of the tests suffering from ceiling effects and low responsiveness. For example, 17 out of 26 (65%) control participants and 17 out of 21 (81%) Yoga participants attained the highest possible standing balance score at follow-up, indicating that this measure suffers from ceiling effects in this population. Additionally, it was unlikely that the Yoga programme would alter usual walking pace over 4 m. Alternative physical function tests, such as the 30-s chair rise, single leg stance, and 8-ft timed up-and-go, could be considered for use in future studies. Nevertheless, the results of this study provide encouraging evidence that the BWY Gentle Years Yoga© programme has several beneficial effects in older adults with various health problems.

High attrition rates have been reported among older exercisers in general [27]. This 10-week adapted Yoga programme showed a relatively low attrition rate of 16%, with the reasons for withdrawal being unrelated to the intervention. The feasibility of the Yoga programme was also demonstrated by the ease of recruitment in a short period of time from a small geographical area, and excellent attendance with two thirds of the participants attending at least 8 of the 10 classes. The interview responses indicated that participants found the programme to be suitable for their abilities and enjoyable. Other factors that likely promoted attendance included the opportunity for social interaction, and friendly and supportive instructors who modified the intervention content to suit individual needs. Although the attendance data may have been biased by the Yoga classes being free of charge, 70% of the Yoga participants had paid to attend the next 10-week course following completion of the 3-month assessment. Finally, the Yoga programme appeared to be safe, as there was only one transient, non-serious adverse event.

Strengths of this study include blinded outcome assessment, low rates of attrition and missing data, and the involvement of multiple teachers (*n* = 7) and intervention fidelity checks. Limitations include the small sample size, short-term follow-up, that the participants were predominantly female (which limits generalisability of the results), and a lack of quantitative data on adherence to the home-based Yoga practice. However, this was designed to be a pilot trial to assess the feasibility of the Yoga programme for older people and to assess the effect on physical function and quality of life in preparation for a larger trial over a wider geographical area. The study has achieved these aims.

## Conclusions

A weekly group-based adapted Yoga programme with home practice appears to be a safe, feasible and acceptable activity for older adults with a broad range of comorbidities, which can lead to improvements in physical function and mental and social well-being. Further research is needed to confirm and expand on these findings.

## Abbreviations

BWY: British Wheel of Yoga
CI: Confidence interval
EQ-5D: EuroQol five dimensions questionnaire
EQ-VAS: EuroQol Visual Analogue Scale
SD: Standard deviation
SPPB: Short Physical Performance Battery
WEMWBS: Warwick-Edinburgh Mental Well-being Scale
